# Global climate change: impact of heat waves under different definitions on daily mortality in Wuhan, China

**DOI:** 10.1186/s41256-017-0030-2

**Published:** 2017-04-05

**Authors:** Yunquan Zhang, Renjie Feng, Ran Wu, Peirong Zhong, Xiaodong Tan, Kai Wu, Lu Ma

**Affiliations:** 1 Department of Epidemiology and Biostatistics, School of Health Sciences, Wuhan University, 185 Donghu Road, Wuchang District, Wuhan, 430071 China; 2 Department of Occupational and Environmental Health, School of Health Sciences, Wuhan University, 185 Donghu Road, Wuchang District, Wuhan, 430071 China; 3 Jiang’an District Center for Disease Control and Prevention, 3 Chezhan Road, Jiang’an District, Wuhan, 430014 China

**Keywords:** Climate change, Temperature, Heat wave, Definition, Mortality, China

## Abstract

**Background:**

There was no consistent definition for heat wave worldwide, while a limited number of studies have compared the mortality effect of heat wave as defined differently. This paper aimed to provide epidemiological evidence for policy makers to determine the most appropriate definition for local heat wave warning systems.

**Methods:**

We developed 45 heat wave definitions (HWs) combining temperature indicators and temperature thresholds with durations. We then assessed the impact of heat waves under various definitions on non-accidental mortality in hot season (May–September) in Wuhan, China during 2003–2010.

**Results:**

Heat waves defined by HW14 (daily mean temperature ≥ 99.0th percentile and duration ≥ 3 days) had the best predictive ability in assessing the mortality effects of heat wave with the relative risk of 1.63 (95% *CI*: 1.43, 1.89) for total mortality. The group-specific mortality risk using official heat wave definition of Chinese Meteorological Administration was much smaller than that using HW14. We also found that women, and the elderly (age ≥ 65) were more susceptible to heat wave effects which were stronger and longer lasting.

**Conclusion:**

These findings suggest that region specific heat wave definitions are crucial and necessary for developing efficient local heat warning systems and for providing evidence for policy makers to protect the vulnerable population.

**Electronic supplementary material:**

The online version of this article (doi:10.1186/s41256-017-0030-2) contains supplementary material, which is available to authorized users.

## Background

Global climate change has become one of the biggest health threats in the 21st century [1]. As increased frequency, intensity, and duration of heat wave events occurred associated with global warming [2–4], impact of heat wave on health has drawn more and more attention worldwide. For instance, California heat wave occurring in 2006 was reported to be associated with approximately 16,166 excess emergency department visits and 1182 excess hospitalizations [5]. And in the summer of 2010, Moscow and Western Russia suffered an unprecedented heat wave both in strength and duration, resulting in 55,000 excess heat-related deaths [6]. From a pathophysiological point of view, heat waves were associated with dehydration, increased blood viscosity and impairment of the endothelial function, which would in return increase the risk for thrombo-embolic diseases and cardiovascular events [7].

Until now, there has been no single, consistent definition of heat waves as people may acclimatize themselves to their local climatic zones [8, 9]. In general, heat waves are defined by (1) temperature indicator (e.g., daily average, maximum, or minimum temperature), (2) temperature threshold (e.g., a relative threshold or an absolute threshold), and (3) heat wave duration [10]. Many previous studies applied several heat wave definitions as sensitivity analyses when assessing the health impact of heat wave [11, 12]. However, heat-related mortality risk estimates varied greatly by different heat wave definitions according to several recent studies [10, 13, 14], which demonstrated the importance of heat wave definitions in predicting health effects of heat waves. Moreover, by using the variance-decomposition method, heat wave definitions were found to attribute to 22.2% of the uncertainty for mortality risks during future heat waves in a recent study conducted in the Eastern United States [15]. Hence, it is of great significance to determine the most appropriate definition for heat wave warning systems based on local epidemiological studies.

People in developing countries are more susceptible to heat-related mortality than developed countries due to limited adaptive capacity and vulnerability [16]. However, most of heat-related epidemiological studies were conducted in developed countries (e.g., USA and European countries) [2, 4]. As the largest developing country, China has experienced a rapid aging of the population in recent years [17], which may also lead to an increased health burden from heat [3]. Nevertheless, only a limited number of studies have explored the adverse health impact of heat wave in China, especially in the inland provinces including some major cities [18].

In this study we assessed the impact of heat waves under various definitions on daily mortality in Wuhan, China during 2003–2010, and aimed to provide epidemiological evidence for policy makers to determine the most appropriate definition for local heat wave warning systems.

## Methods

### Study area and population

Wuhan, the capital of Hubei Province and the largest city in central China, is located in the middle of the Yangtze River Delta, at 29°58´–31°22´ north latitude and 113°41´–115°05´ east longitude. Wuhan has typical subtropical, humid, monsoon climate with a distinct pattern of four seasons. Jiang’an District is one of the seven main central urban districts in Wuhan and was the political, economic, cultural, financial, and information center of Wuhan City. The resident population of Jiang’an District was about 0.68 million in 2010 and urban area was 64.24 km^2^. Known as an oven city in China, Wuhan usually experienced very hot summers, with the highest maximum temperature of 39.6 °C during 2003–2010.

### Data collection

Daily mortality data from January 1, 2003 to December 31, 2010 were obtained from Centre for Disease Control and Prevention of Jiang’an District in Wuhan, China. The causes of death were encoded according to the 10th Revision of the International Classification of Disease (ICD-10) and daily non-accidental death (A00-R99) was collected in the present study. Daily meteorological data during 2003–2010, including daily maximum, mean, minimum temperature and relative humidity were obtained from the China Meteorological Data Sharing Service System (http://data.cma.cn/). Daily air pollution data of particulate matter < 10 μm in aerodynamic diameter (PM_10_), sulfur dioxide (SO_2_), and nitrogen dioxide (NO_2_) were collected from the Wuhan Environmental Monitoring Center. As in previous studies [10, 12], we restricted the study period to the hot season (May–September) when heat waves generally occurred in Wuhan.

### Heat wave definition

In order to determine which heat wave definition is the best to capture the effects on non-accidental mortality in Wuhan, we developed 45 heat wave definitions combining temperature indicators (mean temperature, maximum temperature, and minimum temperature), temperature thresholds [8, 10, 19] (90.0th, 92.5th, 95th, 97.5th, and 99.0th percentile of daily mean/maximum/minimum temperature during 2003–2010) with duration of ≥2, ≥3, and ≥4 days.

### Data analysis

A quasi-Poisson generalized linear model (GLM), which allows for the over-dispersion in daily non-accidental deaths, was used to evaluate the relative risk of mortality on heat-wave days compared with non-heat-wave days in hot season (May–September) [8]. Based on different heat wave definitions, heat wave was categorized as a binary variable, which equalled to 1 for heat-wave days and 0 for non-heat-wave days. Several covariates were incorporated in the GLM: (1) 4 degree of freedom (df) natural cubic spline for day of the year to exclude seasonal trends in daily mortality based on the Akaike Information Criterion for quasi-Poisson, namely Q-AIC (smaller is better); (2) categorical variable for year to control for long-term trend [8]; (3) three df natural cubic spline of relative humidity in accordance with previous studies [20]; (4) indicator variables for “day of the week (DOW)” and public holidays [20]. The GLM model was given as follows:

$$ {Y}_t\sim Poisson\left({u}_t\right) $$

$$ \mathrm{Log}\left({u}_t\right)=\upalpha + ns\left( doy, df=4\right)+ year+ ns\left( Rh, df=3\right)+ DO{W}_t+ H olida{y}_t+ H W s $$

*Y*_*t*_ and *μ*_*t*_ are the observed and expected daily number of non-accidental death on day *t*, respectively. *α* is the intercept, and *ns* refers to natural cubic spline. *doy* and *Rh* mean day of the year and relative humidity, respectively. *HWs* represents the binary variable for heat waves under different definitions.

According to a previous study [8], we assessed the best model fit among the 45 various heat wave definitions by minimizing the sum of the Akaike Information Criterion for quasi-Poisson (Q-AIC) values from all group-specific mortality (total, male, female, age < 65, and age ≥ 65). In addition, we compared the mortality risks of heat wave defined by Chinese Meteorological Administration [21, 22] (HW_CMA_, daily maximum temperature ≥35 °C and duration ≥ 3 days) with the most appropriate definition determined by the above criterion.

To demonstrate the association between mortality and heat wave more comprehensively, we also conducted the lag effect analyses (lag0 to lag10) of heat waves, which separately assessed the mortality impact several days (0 to 10) posterior to heat waves. Mortality effects of heat waves at lag1 day, for instance, were assessed by linking heat wave exposure (i.e. heat-wave day or non-heat-wave day) 1 day prior to deaths with the non-accidental deaths on the current day.

Sensitivity analyses were performed by changing the df (5 to 8) for day of the year to control for seasonality and df (4 to 6) for relative humidity. Besides, we examined the possible confounding effects of air pollutants (i.e., PM_10_, SO_2_, and NO_2_) since short-term exposures to these pollutants were also found associated with daily mortality in numerous epidemiological studies. Additionally, we verified the model fits of different heat wave definitions by using another evaluation standard (Bayesian information criterion for quasi-Poisson, Q-BIC).

All analyses were conducted with R software (version 3.1.3; http://www.r-project.org/). The statistical tests were two-sided, and effects of *P* < 0.05 were considered statistically significant.

## Results

### Descriptive statistics of daily death, meteorological factors and concentrations of air pollutants

Table 1 summarizes the statistic characteristics in hot seasons (May–September) in Wuhan, China during 2003–2010. A total of 11,824 non-accidental deaths occurred during the study period, including 5237 (44.3%) females and 8562 (72.4%) people over 65 years old. The average daily mean temperature was 26.7 °C (range from 12.7 to 35.8 °C), the average daily maximum temperature was 30.9 °C (range from 18.9 to 39.6 °C), and the average daily relative humidity was 71.4% (range from 37 to 96%). The average daily concentration of air pollutants were 90.5 ± 44.0 μg/m^3^, 35.4 ± 19.7 μg/m^3^, and 46.4 ± 18.9 μg/m^3^ for PM_10_, SO_2_, and NO_2_, respectively.

**Table 1:** Descriptive statistics of daily death, meteorological factors and concentrations of air pollutants in hot season (May–September) in Wuhan, China during 2003–2010

Variable	Mean ± SD	Min	*P*_25_	Median	*P*_75_	Max
Daily death						
Total	9.7 ± 3.3	1	7	9	12	34
Male	5.4 ± 2.4	0	4	5	7	16
Female	4.3 ± 2.3	0	3	4	6	21
Age < 65	2.7 ± 1.6	0	1	3	4	10
Age ≥ 65	7.0 ± 2.9	0	5	7	9	31
Meteorological factors						
Minimum temperature (°C)	23.6 ± 3.8	10.8	20.8	23.8	26.3	32.3
Mean temperature (°C)	26.7 ± 3.9	12.7	24.0	26.9	29.7	35.8
Maximum temperature (°C)	30.9 ± 4.2	18.9	28.3	31.3	34.0	39.6
Relative humidity (%)	71.4 ± 10.7	37	64	72	79	96
Air Pollutants (μg/m^3^)						
PM_10_	90.5 ± 44.0	11	58	82	115	337
SO_2_	35.4 ± 19.7	2	21	32	46	133
NO_2_	46.4 ± 18.9	13	33	43	55	288

### Heat wave definitions and model fits using Q-AIC values for different heat wave definitions

Table 2 describes 46 definitions of heat waves defined by daily mean, maximum, and minimum temperature, and calculates the sum of Q-AIC values from all group-specific mortality for different heat wave definitions. Heat waves (HW14, HW29, and HW43) defined by threshold of 99.0th percentile of temperature distribution with duration ≥ 3 days, ≥3 days, and ≥2 days gave the lowest Q-AIC value (27627.63, 27658.92, and 27641.87) when using daily mean, maximum, and minimum temperature as the temperature indicator, respectively. And the model using HW_CMA_ as heat wave definition produced the Q-AIC value of 27726.94. Moreover, HW14 defined by daily mean temperature ≥ 33.3 °C (99th percentile) with duration ≥ 3 days performed best in model fits among the 46 heat wave definitions.

**Table 2:** The 46 heat wave definitions (HW01-HW45, and HW_CMA_) and the sum of Q-AIC values from all group-specific mortality for different heat wave definitions in hot season (May–September) in Wuhan, China during 2003–2010

Temperature indicator	Temperature threshold	Definitions and Q-AIC values		
		Duration ≥ 2 days	Duration ≥ 3 days	Duration ≥ 4 days
Mean temperature	P_90.0_ (29.8 °C)	HW01 (27760.40)	HW02 (27762.54)	HW03 (27767.14)
	P_92.5_ (30.7 °C)	HW04 (27759.36)	HW05 (27770.44)	HW06 (27753.90)
	P_95.0_ (31.7 °C)	HW07 (27755.96)	HW08 (27757.35)	HW09 (27737.22)
	P_97.5_ (32.6 °C)	HW10 (27707.33)	HW11 (27672.28)	HW12 (27684.47)
	P_99.0_ (33.3 °C)	HW13 (27666.18)	HW14 (27627.63)	HW15 (27650.23)
Maximum temperature	P_90.0_ (34.2 °C)	HW16 (27749.33)	HW17 (27742.48)	HW18 (27758.81)
	P_92.5_ (35.2 °C)	HW19 (27750.06)	HW20 (27744.90)	HW21 (27724.81)
	P_95.0_ (35.9 °C)	HW22 (27743.21)	HW23 (27736.82)	HW24 (27705.14)
	P_97.5_ (36.7 °C)	HW25 (27750.16)	HW26 (27724.53)	HW27 (27678.12)
	P_99.0_ (37.4 °C)	HW28 (27690.82)	HW29 (27658.92)	HW30 (27685.73)
	35 °C		HW_CMA_ (27726.94)	
Minimum temperature	P_90.0_ (26.5 °C)	HW31 (27775.58)	HW32 (27778.37)	HW33 (27768.97)
	P_92.5_ (27.3 °C)	HW34 (27785.54)	HW35 (27780.29)	HW36 (27774.44)
	P_95.0_ (28.3 °C)	HW37 (27755.26)	HW38 (27739.75)	HW39 (27735.15)
	P_97.5_ (29.3 °C)	HW40 (27714.22)	HW41 (27689.72)	HW42 (27696.01)
	P_99.0_ (30.2 °C)	HW43 (27641.87)	HW44 (27653.93)	HW45 (27653.93)

Additional file 1: Table S1 shows that the number of heat-wave days and daily deaths during 2003–2010 in Wuhan under 45 different heat wave definitions. Similar number of heat-wave days and daily deaths were identified using daily maximum/minimum temperature metric and daily mean temperature metric in heat wave definitions. Less heat-wave days and more daily deaths on heat-wave days occurred when heat waves were defined by higher percentile of temperature thresholds and longer durations, while daily deaths on non-heat-wave days changed little along with heat wave definitions.

### Mortality effects of heat wave under 45 different definitions

**Fig. 1:**
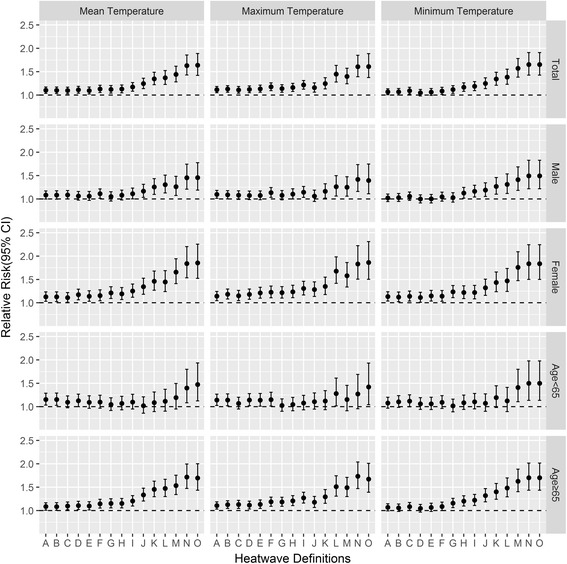
Relative risk of group-specific mortality on heat-wave days compared with non-heat-wave days by 45 heat wave definitions in hot season (May to September) in Wuhan, China during 2003–2010. Heat wave definitions of A to O defined by mean temperature (*Left*) referred to HW01 to HW15, definitions of A to O defined by maximum temperature (*Middle*) referred to HW16 to HW30, and definitions of A to O defined by minimum temperature (*Right*) referred to HW31 to HW45, respectively

Figure 1 presents the mortality effects of heat wave using 45 different heat wave definitions. Group-specific mortality risks varied greatly by different heat wave definitions. For instance, heat waves using HW01 and HW14 were associated with the relative risks of 1.10 (95% *CI*: 1.04, 1.17) and 1.63 (95% *CI*: 1.43, 1.89) for total mortality, respectively, 1.08 (95% *CI*: 1.00, 1.17) and 1.45 (95% *CI*: 1.21, 1.74) for male mortality, 1.13 (95% *CI*: 1.03, 1.24) and 1.84 (95% *CI*: 1.54, 2.20) for female mortality, 1.15 (95% *CI*: 1.03, 1.29) and 1.40 (95% *CI* : 1.09, 1.80) for mortality of people age < 65, 1.08 (95% *CI*: 1.01, 1.17) and 1.71 (95% *CI*: 1.47, 2.00) for mortality of people age ≥ 65. Similar results were also found using daily maximum and minimum temperature metrics and daily mean temperature metric.

### Group-specific mortality effects of heat wave using HW14, HW29, HW43, and HW_CMA_

Table 3 shows the group-specific mortality effects of heat wave using definitions of HW14, HW29, HW43, and HW_CMA_. The results of mortality risk using heat wave definitions of HW14, HW29, and HW43 were comparable, while heat wave defined by HW29 was not significantly associated with mortality for people younger than 65. However, when using official heat wave definition by Chinese Meteorological Administration, the estimated effects of heat wave on mortality became weakened when compared with those using definitions of HW14, HW29, and HW43. Relative risks of mortality using HW_CMA_ were 1.15 (95% *CI*: 1.08, 1.23) for the total population, 1.10 (95% *CI*: 1.01, 1.19) for males, 1.23 (95% *CI*: 1.12, 1.35) for females, 1.12 (95% *CI*: 1.00, 1.26) for people younger than 65, and 1.16 (95% *CI*: 1.08, 1.26) for people of age ≥65. Generally, females and elderly people were more susceptible to the mortality risk of heat waves by using different heat wave definitions. Moreover, associations between heat waves and daily mortality almost kept unchanged with and without adjusting for air pollutants (PM_10_, SO_2_, and NO_2_).

**Table 3:** Relative risk of group-specific mortality on heat-wave days compared with non-heat-wave days (using heat wave definition HW14, HW29, HW43, and HW_CMA_) with and without adjusting for air pollutants in Wuhan, China during 2003–2010

Heat wave definition	Subgroups	Unadjusted		+PM_10_		+SO_2_		+NO_2_	
		RR	95% CI	RR	95% CI	RR	95% CI	RR	95% CI
HW14	Total	1.63	(1.43,1.86)	1.63	(1.43,1.86)	1.63	(1.43,1.86)	1.63	(1.43,1.86)
	Male	1.45	(1.21,1.74)	1.45	(1.21,1.75)	1.46	(1.21,1.75)	1.45	(1.21,1.75)
	Female	1.84	(1.54,2.20)	1.84	(1.54,2.21)	1.85	(1.54,2.21)	1.84	(1.54,2.21)
	Age < 65	1.4	(1.09,1.80)	1.4	(1.09,1.80)	1.4	(1.09,1.81)	1.4	(1.09,1.80)
	Age ≥ 65	1.71	(1.47,2.00)	1.71	(1.47,2.00)	1.72	(1.47,2.00)	1.72	(1.47,2.00)
HW29	Total	1.61	(1.40,1.85)	1.61	(1.39,1.85)	1.61	(1.40,1.86)	1.61	(1.40,1.85)
	Male	1.42	(1.16,1.74)	1.42	(1.16,1.73)	1.42	(1.16,1.74)	1.42	(1.16,1.74)
	Female	1.83	(1.51,2.22)	1.83	(1.51,2.22)	1.83	(1.51,2.23)	1.83	(1.51,2.23)
	Age < 65	1.27	(0.96,1.69)	1.27	(0.96,1.69)	1.28	(0.96,1.70)	1.27	(0.96,1.69)
	Age ≥ 65	1.73	(1.47,2.04)	1.73	(1.47,2.04)	1.73	(1.47,2.04)	1.73	(1.47,2.04)
HW43	Total	1.57	(1.39,1.78)	1.59	(1.40,1.80)	1.58	(1.39,1.79)	1.58	(1.39,1.79)
	Male	1.41	(1.18,1.69)	1.42	(1.19,1.70)	1.42	(1.19,1.69)	1.41	(1.18,1.69)
	Female	1.76	(1.48,2.09)	1.78	(1.49,2.12)	1.77	(1.48,2.10)	1.77	(1.48,2.10)
	Age < 65	1.41	(1.11,1.80)	1.41	(1.11,1.80)	1.42	(1.11,1.81)	1.41	(1.11,1.80)
	Age ≥ 65	1.63	(1.40,1.89)	1.65	(1.42,1.91)	1.63	(1.41,1.89)	1.63	(1.41,1.89)
HW_CMA_	Total	1.15	(1.08,1.23)	1.16	(1.08,1.23)	1.15	(1.08,1.23)	1.15	(1.08,1.23)
	Male	1.10	(1.01,1.19)	1.10	(1.01,1.19)	1.10	(1.01,1.19)	1.10	(1.01,1.19)
	Female	1.23	(1.12,1.35)	1.23	(1.12,1.35)	1.23	(1.12,1.35)	1.23	(1.12,1.35)
	Age < 65	1.12	(1.00,1.26)	1.12	(1.00,1.26)	1.13	(1.00,1.26)	1.13	(1.00,1.26)
	Age ≥ 65	1.16	(1.08,1.26)	1.17	(1.08,1.26)	1.17	(1.08,1.26)	1.16	(1.08,1.26)

### Lag patterns and group-specific mortality effects of heat wave using HW14 and HW_CMA_

**Fig. 2:**
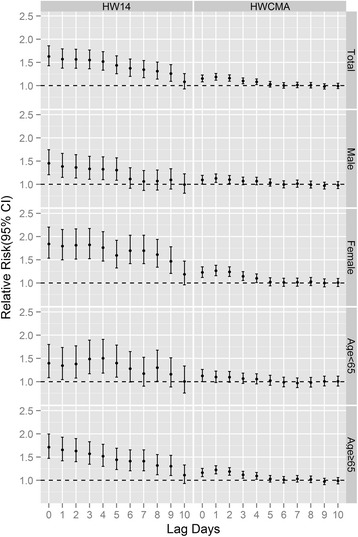
Relative risk of mortality on different lag days on heat-wave days compared with non-heat-wave days using HW14 and HW_CMA_ in hot season (May to September) in Wuhan, China during 2003–2010

Figure 2 presents the lag patterns of heat wave effects on group-specific mortality using HW14 and HW_CMA_. Generally, for total mortality, the highest mortality risk appeared on the first day (lag0) when heat waves occurred and the effect of heat waves persisted for several days’ duration (usually less than 10 days). Lag characteristics of mortality impact varied greatly by using different heat wave definitions, in spite of the similar tendency of gradual decay in the mortality risk. The heat wave effects were much stronger and longer lasting using definition of HW14 compared with HW_CMA_. Additionally, the lag patterns of heat wave effects were modified by different genders and age groups. Females and the elderly (age ≥65) were more vulnerable to the mortality impact of heat waves using both HW14 and HW_CMA_ with higher mortality risks and longer lasting effects.

Our sensitivity analyses indicated that estimated mortality risks were robust when changing the df (5 to 8) for day of the year (Additional file 1: Figure S1) and df (4 to 6) for relative humidity (Additional file 1: Figure S2). Associations between heat waves and daily mortality almost kept unchanged with and without adjusting for air pollutants (Table 3). Similar model fits were consistently observed by using Q-BIC as the evaluation standard, which also determined HW14 as the best predictive ability in assessing the mortality effects of heat wave (Additional file 1: Table S2).

## Discussion

In this study, we evaluated which of the 46 heat wave definitions can best capture the heat wave impact on non-accidental mortality in Wuhan during 2003 to 2010. Compared with non-heat-wave days, heat waves defined by HW14 (daily mean temperature ≥ 99th percentile and duration ≥ 3 days) performed best in predicting the effects of heat wave on group-specific mortality. The estimated mortality effects of heat waves almost kept unchanged with and without adjusting for air pollutants (PM_10_, SO_2_, and NO_2_). We also found that females and the elderly were more susceptible to heat wave effects which were stronger and longer lasting. These findings may have important implications for public health policies to protect people from extremely hot temperatures in Wuhan, China.

Our results found that heat waves were associated with increased daily mortality in Wuhan, which were consistent with several previous studies [23–26]. However, the heat-related mortality in different regions varied greatly [27]. National-level analyses conducted in 66 Chinese communities [20] and 43 U.S. communities [28] showed that the morality risks of heat wave were spatially heterogeneous with greater effects in the northern regions and smaller in the southern regions. The differences revealed that population susceptibility to heat wave may be discrepant due to acclimatization to local climate characteristics through long-term physiological and behavioral adaptation [20, 29]. In addition, socioeconomic factors such as average income, health care system, population density and numbers of household-owned air conditionings were found to modify the health impact of heat wave [30, 31].

A number of previous studies revealed that gender and age might modify the associations between environmental risk factors (such as air pollution, extremely temperature events) and daily mortality [25, 32–34]. In the present study, we found that the elderly were more vulnerable to heat wave-related mortality, which might be due to the reduced thermoregulatory capacity, the status of on medication that may interfere with normal sweating process, low risk perception and adaptation ability to heat wave [12, 20]. The impact of heat wave may become a great health and social burden in the next few decades in China due to the rapid population aging, which demonstrated the importance and urgency for decision makers and the public to design adaptation plan to heat wave for elderly people [20]. Consistent with most studies focusing on temperature-related mortality [1, 2, 12], our results showed a stronger association in females than that in males between heat wave and mortality. However, this finding was less consistent with several other studies [23, 35].

In China, heat wave was nationwide defined as daily maximum temperature ≥35 °C with duration ≥3 days by Chinese Meteorological Administration (CMA). However, some studies indicated that it might be inappropriate for a large country like China to use an absolute temperature as the threshold for defining heat waves due to the variable vulnerability to heat waves in different regions [10, 20]. The health impact of heat wave under different definitions had been examined in two previous studies conducted in Beijing [8] and Nanjing, China [10]. Tian et al. compared 18 heat wave definitions by combining heat wave thresholds (87.5th, 90.0th, 92.5th, 95th, 97.5th, and 99th percentile of daily mean temperature) with different duration days (≥2 to ≥ 4 days) to assess the short-term impact of heat waves on CHD mortality, and found that heat wave definition using 97.5th percentile of daily mean temperature and duration ≥2 days produced the best model fit [8] . Chen et al. reported that heat waves defined as ≥4 consecutive days with daily mean temperature >98th percentile were the most appropriate to estimate the influence of the added effect of heat waves on cause-specific mortality in Nanjing among 15 heat wave definitions [10]. In our study, heat wave defined by HW14 (daily mean temperature >99th percentile and duration ≥3 days) showed the best predictive ability in evaluating heat wave-mortality relationship, and using the CMA definition underestimated the mortality risks of heat waves in Wuhan. Therefore, region specific definitions based on relative temperature thresholds are needed to design effective local heat warning systems [10]. In addition, our results showed that, with the same relative thresholds and durations, heat waves using daily maximum/minimum temperature metrics and daily mean temperature metric had similar estimates of mortality risks, which was consistent with previous studies focusing on temperature-mortality relationship [36, 37]. However, daily mean temperature can capture heat wave effects more completely in the present study, and mean temperature was most commonly used to assess the association between temperature and mortality since mean temperature can represent the exposure throughout the whole day and night and provide more easily interpreted results within a policy context [2, 38].

Previously, a positive association between ambient pollutants and daily mortality has been clearly demonstrated in numerous epidemiological studies. In the present study, we observed that heat wave effects on mortality remained similar with and without adjusting for air pollutants (PM_10_, SO_2_, and NO_2_), even though the concentrations of air pollutants were well above the international health-based standards. Consistent results were also obtained in two recent Chinese studies, one of which was conducted in Nanjing using 18 different definitions of heat wave [10]. The other was conducted in four communities of Guangdong Province using daily API (Air Pollution Index) as the substitutive indicator of air pollution [12]. These studies provided implications for the future researchers that air pollutants would not significantly change the estimated mortality risk of temperature, thus we can also approximately assess the temperature-related health effects without availability of air pollution data.

Our study has several limitations. Firstly, the daily mortality data were obtained from only one district of Wuhan City, which may not completely capture the effects of heat wave in the whole city. Secondly, we did not consider the possible interactions between air pollution and high temperature in the analyses, which might result in overestimated heat wave effects if air pollution and high temperature had synergistic effects on mortality [12]. Thirdly, a number of other thermal indicators, such as ambient apparent temperature, could also be used to define the heat wave. However, we only included daily mean, maximum, and minimum temperature in the present study considering that these are most commonly used heat-related indicators, and can be easily understood by the public. In addition, the influence of ozone on mortality was not included in the analyses due to the unavailability of ozone data. However, the present and previous studies showed that heat-related mortality effects were robust after adjusting for air pollution [10, 39].

## Conclusions

Our study demonstrated a significant increase in mortality during heat wave in Wuhan, China, while the mortality effects of heat waves varied greatly by different heat wave definitions. It was suggested to use daily mean temperature ≥ 33.3 °C (99th percentile) with duration ≥3 days as heat wave definition in Wuhan, as it could best capture the mortality effects of heat wave. Our results also showed that the elderly and females were more vulnerable to the mortality impact of heat waves. These findings suggest that region specific heat wave definitions are crucial and necessary for developing efficient local heat warning systems and providing evidence for policy makers to protect the vulnerable individuals.

## Additional file

Additional file 1: Table S1. Heat-wave days and daily death under 45 heat wave definitions in hot season (May–September) in Wuhan, China during 2003–2010. **Table S2.** The 46 heat wave definitions (HW01-HW45, and HW_CMA_) and the sum of Q-BIC values from all group-specific mortality for different heat wave definitions in hot season (May–September) in Wuhan, China during 2003–2010. **Figure S1.** Sensitive analyses by changing df for day of the year from 4 to 8 (the effect of heat waves on group-specific mortality using definitions of HW14, HW29, HW43, and HW_CMA_). **Figure S2.** Sensitive analyses by changing df for relative humidity from 3 to 6 (the effect of heat waves on group-specific mortality using definitions of HW14, HW29, HW43, and HW_CMA_). (DOCX 877 kb)

## Abbreviations

CI: Confidential interval
CMA: Chinese Meteorological Administration
df: Degree of freedom
DOW: Day of the week
GLM: Generalized linear model
HWs: Heat wave definitions
ICD-10: The 10th Revision of the International Classification of Disease
NO_2_: Nitrogen dioxide
PM_10_: Particulate matter < 10 μm in aerodynamic diameter
Q-AIC: Akaike Information Criterion for quasi-Poisson
SO_2_: Sulfur dioxide
