# Supplementary material for: Global climate change: impact of heat waves under different definitions on daily mortality in Wuhan, China
Source: Glob Health Res Policy. 2017 Apr 5;2:10. doi: 10.1186/s41256-017-0030-2 (PMC5683448; doi:10.1186/s41256-017-0030-2)
Supplement: Additional file 1: Table S1. — Heat-wave days and daily death under 45 heat wave definitions in hot season (May–September) in Wuhan, China during 2003–2010. Table S2. The 46 heat wave definitions (HW01-HW45, and HWCMA) and the sum of Q-BIC values from all group-specific mortality for different heat wave definitions in hot season (May–September) in Wuhan, China during 2003–2010. Figure S1. Sensitive analyses by changing df for day of the year from 4 to 8 (the effect of heat waves on group-specific mortality using definitions of HW14, HW29, HW43, and HWCMA). Figure S2. Sensitive analyses by changing df for relative humidity from 3 to 6 (the effect of heat waves on group-specific mortality using definitions of HW14, HW29, HW43, and HWCMA). (DOCX 877 kb) [file 41256_2017_30_MOESM1_ESM.docx]

**Supplemental Material**

Global climate change: Impact of heat waves under different definitions on daily mortality in Wuhan, China

Yunquan Zhang ^1^, Renjie Feng ^1^,Ran Wu ^1^, Peirong Zhong ^1^, Xiaodong Tan ^2^, Kai Wu ^3,*^, Lu Ma ^1,*^

^1^ Department of Epidemiology and Biostatistics, School of Health Sciences, Wuhan University, 185 Donghu Road, Wuchang District, Wuhan 430071, China; E-mails: [Yun-quanZhang@whu.edu.cn](mailto:Yun-quanZhang@whu.edu.cn)(Y.Z.); 2014203050012@whu.edu.cn (R.F.); wuran@whu.edu.cn (R.W.); zhongpr_1993@126.com (P.Z.);

^2^ Department of Occupational and Environmental Health, School of Health Sciences,Wuhan University, 185 Donghu Road, Wuchang District, Wuhan 430071, China; E-mail: xiaodongtan@yahoo.com (X.T.);

^3^ Jiang’an District Center for Disease Control and Prevention, 3 Chezhan Road, Jiang’an District, Wuhan 430014, China; E-mail: uueng@hotmail.com (K.W.)

* Corresponding author:

E-mail: [uueng@hotmail.com](mailto:uueng@hotmail.com) (Kai Wu); [malu@whu.edu.cn (Lu](mailto:malu@whu.edu.cn%20(Lu) Ma)

**Table S1** Heat-wave days and daily death under 45 heat wave definitions in hot season (May–September) in Wuhan, China during 2003–2010.

| Heat wave  definitions ^a^ | N. of heat-wave days | Daily death | | Heat wave  definitions ^b^ | N. of heat-wave days | Daily death | | Heat wave  definitions ^c^ | N. of heat-wave days | Daily death | |
| --- | --- | --- | --- | --- | --- | --- | --- | --- | --- | --- | --- |
|  |  | Heat-wave days | Non-heat-wave days |  |  | Heat-wave days | Non-heat-wave days |  |  | Heat-wave days | Non-heat-wave days |
| HW01 | 270 | 10.5±3.8 | 9.4±3.2 | HW16 | 269 | 10.5±3.8 | 9.4±3.2 | HW31 | 274 | 10.3±3.6 | 9.5±3.2 |
| HW02 | 250 | 10.5±3.8 | 9.5±3.2 | HW17 | 237 | 10.6±3.8 | 9.4±3.2 | HW32 | 236 | 10.4±3.7 | 9.5±3.2 |
| HW03 | 217 | 10.5±3.9 | 9.5±3.2 | HW18 | 204 | 10.6±3.9 | 9.5±3.2 | HW33 | 206 | 10.5±3.9 | 9.5±3.2 |
| HW04 | 201 | 10.6±3.9 | 9.5±3.2 | HW19 | 196 | 10.7±3.8 | 9.5±3.2 | HW34 | 199 | 10.3±3.9 | 9.5±3.2 |
| HW05 | 181 | 10.6±3.9 | 9.5±3.2 | HW20 | 172 | 10.8±4.0 | 9.5±3.2 | HW35 | 171 | 10.5±4.0 | 9.5±3.2 |
| HW06 | 160 | 10.8±4.0 | 9.5±3.2 | HW21 | 139 | 11.2±4.2 | 9.5±3.2 | HW36 | 153 | 10.7±4.1 | 9.5±3.2 |
| HW07 | 140 | 10.9±4.2 | 9.5±3.2 | HW22 | 130 | 11.0±4.2 | 9.5±3.2 | HW37 | 129 | 10.9±4.1 | 9.5±3.2 |
| HW08 | 128 | 11.0±4.3 | 9.5±3.2 | HW23 | 108 | 11.3±4.4 | 9.5±3.2 | HW38 | 105 | 11.3±4.3 | 9.5±3.2 |
| HW09 | 98 | 11.5±4.5 | 9.5±3.2 | HW24 | 96 | 11.7±4.4 | 9.5±3.2 | HW39 | 87 | 11.6±4.6 | 9.5±3.2 |
| HW10 | 62 | 12.0±5.1 | 9.5±3.2 | HW25 | 70 | 11.6±5.0 | 9.5±3.2 | HW40 | 58 | 12.0±5.2 | 9.5±3.2 |
| HW11 | 44 | 13.0±5.3 | 9.5±3.2 | HW26 | 48 | 12.4±5.5 | 9.6±3.2 | HW41 | 40 | 12.9±5.4 | 9.6±3.2 |
| HW12 | 35 | 13.3±5.3 | 9.6±3.2 | HW27 | 24 | 14.3±6.3 | 9.6±3.2 | HW42 | 28 | 13.6±6.2 | 9.6±3.2 |
| HW13 | 27 | 14.3±6.0 | 9.6±3.2 | HW28 | 25 | 14.2±6.3 | 9.6±3.2 | HW43 | 21 | 15.2±6.0 | 9.6±3.2 |
| HW14 | 19 | 16.1±6.0 | 9.6±3.2 | HW29 | 15 | 16.2±7.4 | 9.6±3.2 | HW44 | 15 | 16.3±6.7 | 9.6±3.2 |
| HW15 | 16 | 16.1±6.5 | 9.6±3.2 | HW30 | 12 | 16.1±7.5 | 9.6±3.2 | HW45 | 15 | 16.3±6.7 | 9.6±3.2 |

Notes: ^a^ Definitions based on mean temperature; ^b^ Definitions based on maximum temperature; ^c^ Definitions based on minimum temperature.

**Table S2** The 46 heat wave definitions (HW01-HW45, and HW_CMA_) and the sum of Q-BIC values from all group-specific mortality for different heat wave definitions in hot season (May–September) in Wuhan, China during 2003–2010.

| Temperature indicator | Temperature threshold | Definitions and Q-BIC values | | |
| --- | --- | --- | --- | --- |
|  |  | Duration≥2 days | Duration≥3 days | Duration≥4 days |
| Mean temperature | P_90.0_(29.8°C) | HW01(28388.78) | HW02 (28391.39) | HW03 (28396.27) |
|  | P_92.5_(30.7°C) | HW04 (28387.73) | HW05 (28399.83) | HW06 (28381.49) |
|  | P_95.0_(31.7°C) | HW07(28383.55) | HW08 (28384.81) | HW09 (28362.06) |
|  | P_97.5_(32.6°C) | HW10 (28327.61) | HW11 (28288.43) | HW12 (28301.78) |
|  | P_99.0_(33.3°C) | HW13 (28281.66) | **HW14 (28239.02)** | HW15 (28263.77) |
| Maximum temperature | P_90.0_(34.2°C) | HW16 (28376.61) | HW17 (28369.17) | HW18 (28386.99) |
|  | P_92.5_(35.2°C) | HW19 (28377.59) | HW20 (28371.65) | HW21 (28348.84) |
|  | P_95.0_(35.9°C) | HW22 (28369.58) | HW23 (28362.03) | HW24 (28327.04) |
|  | P_97.5_(36.7°C) | HW25 (28376.19) | HW26 (28347.09) | HW27 (28294.86) |
|  | P_99.0_(37.4°C) | HW28 (28308.7) | HW29 (28272.88) | HW30 (28284.32) |
|  | 35°C |  | HW_CMA_(28351.87) |  |
| Minimum temperature | P_90.0_(26.5°C) | HW31(28406.41) | HW32(28409.43) | HW33(28398.99) |
|  | P_92.5_(27.3°C) | HW34(28416.69) | HW35(28410.78) | HW36(28404.25) |
|  | P_95.0_(28.3°C) | HW37(28382.98) | HW38(28365.23) | HW39(28359.82) |
|  | P_97.5_(29.3°C) | HW40(28335.04) | HW41(28307.99) | HW42(28314.01) |
|  | P_99.0_(30.2°C) | HW43(28255.02) | HW44(28267.67) | HW45(28267.67) |


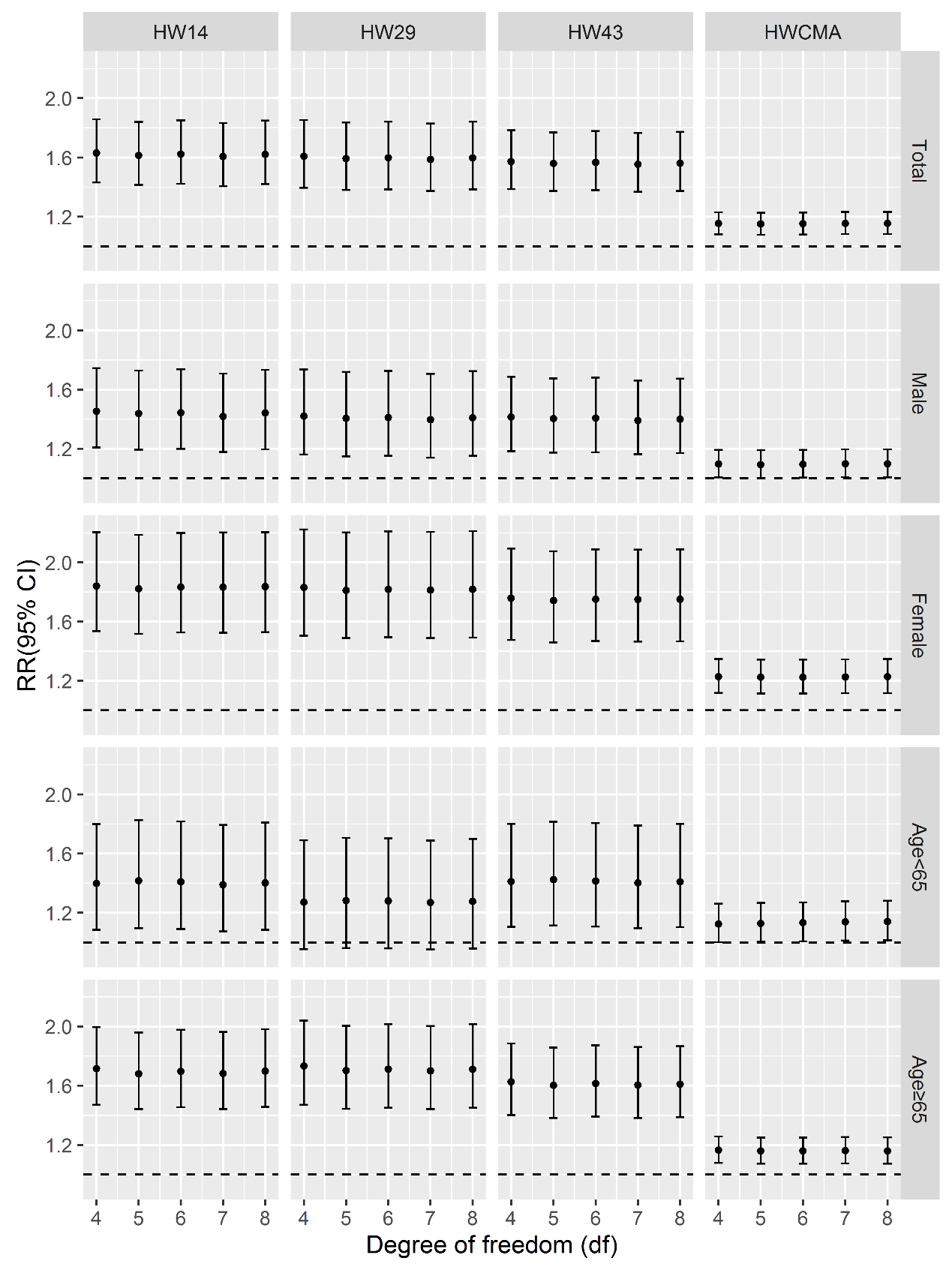


**Figure S1** Sensitive analyses by changing df for day of the year from 4 to 8 (the effect of heat waves on group-specific mortality using definitions of HW14, HW29, HW43, and HW_CMA_)


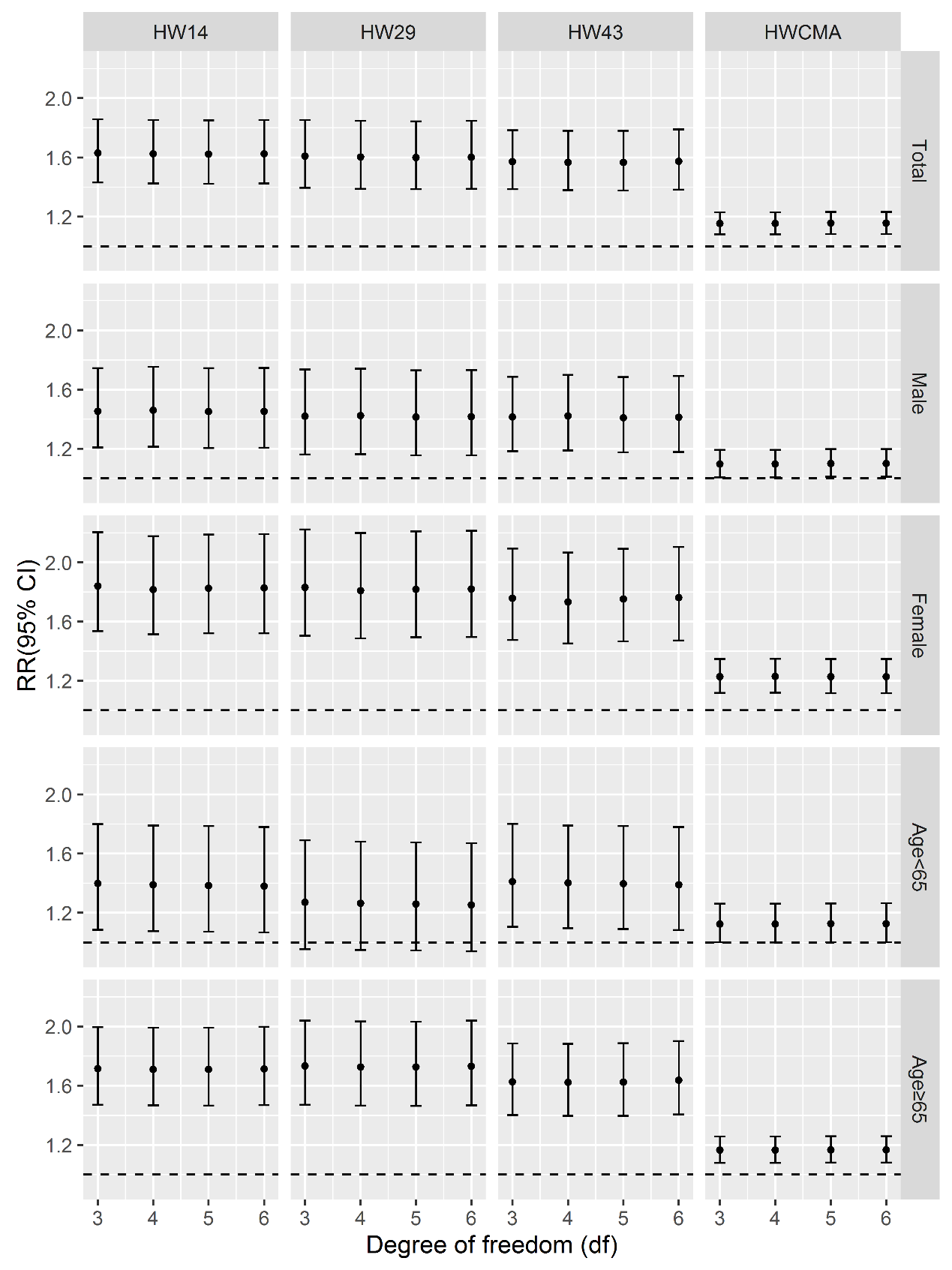


**Figure S2** Sensitive analyses by changing df for relative humidity from 3 to 6 (the effect of heat waves on group-specific mortality using definitions of HW14, HW29, HW43, and HW_CMA_)
